# Implementation and outcomes in benign gynecological surgery with HUGO™ RAS system 12 months initial experience

**DOI:** 10.1007/s11701-024-02109-z

**Published:** 2024-09-26

**Authors:** Yael Yagur, Martin A. Martino, Mikhail Sarofim, Mohammed Almoqren, Hayley Anderson, Jessica Robertson, Sarah Choi, David Rosen, Danny Chou

**Affiliations:** 1 Sydney Women’s Endosurgery Centre (SWEC), St George Hospital, Kogarah, Sydney, NSW Australia; 2 University of New South Wales, Sydney, NSW Australia; 3 Department of Obstetrics and Gynecology, Meir Medical Center, Kfar Saba, affiliated With School of Medicine, Faculty of Medical and Health Sciences, Tel Aviv University, Tel Aviv, Israel; 4 Ascension St. Vincent’s, Jacksonville, Florida, University of South Florida, Tampa, Florida USA

**Keywords:** Robotic assisted surgery, Hugo™, Da Vinci, Learning curve, Port placement, Docking time

## Abstract

We share our experience with the Hugo™ Robotic-Assisted Surgery system in benign gynecological surgeries. We retrospectively analyzed patients who underwent elective robotic surgeries for benign gynecological conditions at our surgical center from February 2023 to February 2024. Data collected included patient demographics, surgery indications, and outcomes. Perioperative data on port-placement time, arm configurations, docking, and console time were documented. Procedural outcome data including troubleshooting and overall satisfaction were also recorded. The primary outcome was perioperative data on port placement, docking time, arm configuration, and console time. The secondary outcome was defined as team satisfaction, system troubleshooting, arm repositioning, and complications graded 3–4 on the Clavien–Dindo Scale. A total of 60 patients underwent procedures for benign gynecological conditions using the Hugo™ RAS over the 12-month study period, primarily for pelvic endometriosis (53%), hysterectomies (27%), and adnexal surgery (10%). The mean port-placement time was 13 min and 41 s. In 31% of cases, low-port placement was used, with arm positioning being asymmetrical in 63% and symmetrical in 37%, demonstrating the system’s flexibility in customizing port configurations while optimizing cosmetic outcomes. Docking time averaged 5 min and 51 s, and console time was 1 h and 5 min. Operational challenges included arm tremors and limited workspace for the assistant. This study details our knowledge using the Hugo™ RAS. Learning curves of port placement, arm positioning, docking, and procedure time can be rapidly adapted in a well-trained team. Our experience suggests the technology is still in its learning curve period.

## Introduction

Minimally invasive gynecological surgery has emerged as the main approach in the realm of benign gynecological procedures, with well-documented benefits. These advantages include a reduction in postoperative pain, complication rates, and hospitalization durations while concurrently facilitating an accelerated recovery period when compared with traditional laparotomy. [1].

Over the past 20 years, robotic-assisted laparoscopic surgery has shifted significantly from traditional laparoscopic surgeries. In just two years, the use of robotic-assisted laparoscopic surgery has increased by 36% [2] for common gynecological procedures including hysterectomy, myomectomy, sacrocolpopexy and management of severe endometriosis in Europe and the United States [3–6].

Robotic surgeries are designed with the potential to offer advantages, and have been more established and researched, particularly on the da Vinci platform (Intuitive, United states) [7]. These include the use of three-dimensional vision as opposed to the traditional two-dimensional view, higher magnification, tremor filtration, improved articulation with a wider range of motion, and enhanced ergonomic design [8]. These innovative features have substantially elevated precision, accuracy, and efficiency, especially in complex surgical procedures, potentially leading to reduced complications and shorter recovery times [9–11]. However, it is important to acknowledge that despite its numerous advantages, robotic surgery is not without limitations, such as higher costs and longer operative times [6].

The Hugo™ RAS system (Medtronic, United states) introduced in 2022 is distinguished by its concept of independent robotic arms, akin to the Versius™ (Cambridge Medical, UK) robot [12], Hugo™ offers an enhanced configuration flexibility and an open console with diverse gripping techniques. However, our understanding of the optimal utilization of Hugo™ is still in its nascent stages, and we are in the process of gaining insights into harnessing its full potential [13].

The majority of data related to the system, from a gynecological perspective, have been presented through case reports or case series, with a particular focus on key parameters, such as docking time, operative time, and major safety events [14, 15]. One of the most extensive study conducted on this system involved 192 cases and aimed to address previously unexplored aspects of the learning curve associated with docking and operation time. Their principal finding indicated that these procedures can be performed efficiently in terms of time, and the specific robotic learning curve appears to be consistent with the existing data for other platforms [16].

The Hugo™ RAS, while promising, still requires testing across essential parameters to provide guidance for newcomers. In this manuscript, we share our experiences and insights through the utilization of the Hugo™ RAS system. Our focus was on the critical aspects of port placement, arm positioning, and docking time. The study was conducted by the same team, which has undergone training for the Hugo™ RAS. The primary robotic surgeon has experience with over a thousand cases using the Da Vinci robotic system prior to study period. We aim to describe the broader surgical experience, with the goal of establishing Hugo™ RAS as a versatile and efficacious platform applicable to various benign gynecological procedures.

## Methods

### Patients

This retrospective observational study included all patients who underwent elective robotic surgeries using the Hugo™ RAS system for benign gynecological conditions between February 2023 and February 2024.

Patients aged between 18 and 65 years were included. The procedures were performed by a single robotic surgeon and consistent robotic team, all of whom had undergone robotic training as described later. The procedures included elective robotic cases of endometriosis resection (stages I-IV, including rectal shaving), hysterectomy (with or without salpingectomy or oophorectomy), myomectomy, cervical cerclage, and cesarean section scar defect repair. These procedures were chosen based on clinical assessments, physical examinations, and sonographic evaluations in accordance with the established and accepted medical criteria. All surgeries that fell under the previously described categories were included without exclusion.

### Outcomes

The primary outcomes were perioperative port placement, docking time, arm configuration, and console time. Secondary outcomes were defined as team satisfaction, system troubleshooting, arm repositioning, and complications at Clavien–Dindo Scale grade 3–4.

### Data

Data were collected for this study from electronic medical records, including patient demographics, medical history, indication for surgery, surgical findings and reports, and a two-month postoperative follow-up. Specific perioperative data were recorded with respect to time from patient entrance to first skin incision, port placement, arm configurations, time from the first incision to port insertion, duration of docking up to console time, and whole surgery time (console time). In addition, information was documented regarding procedural outcome data, including troubleshooting, arm repositioning, instances of arm clashes, instrument breakages, and overall satisfaction of the surgical team.

### Ethical considerations

This study was conducted in accordance with the principles of the Declaration of Helsinki. All methods were performed in accordance with relevant guidelines and regulations. All experimental protocols were approved by the Ethics Institutional Review Board #2023/ETH02370. Given the observational nature of this study, informed consent was not required.

### Robotic surgical team

Elective gynecological surgeries for benign conditions were conducted using the Hugo™ RAS system, led by a minimally invasive gynecology specialist with more than 1200 procedures using the Da Vinci robotic system prior to the study period and a team of anesthetists, surgical fellows, and nurses who have been trained together in robotic surgery.

### Robotic training program

All team members as described above completed the Hugo™ RAS Ascend training program which included three stages: 1. introduction phase: Hugo™ demonstration, e-learning modules and practice on a virtual simulator. 2. Hands-on training: technical training, dry lab exercises, and collaborative training with the entire theater team. 3. Proctored Case: live case observation, video case review and continuous support to ensure competence in utilizing the system.

### Robotic surgery

Various time-related events have also been documented. These events included the time from entering the operating theater to making the first incision, initiation of port placement, docking time (including instrument insertion), console time, skin closure, dressing time, and the moment when the patient left the operating theater.

**Fig. 1:**
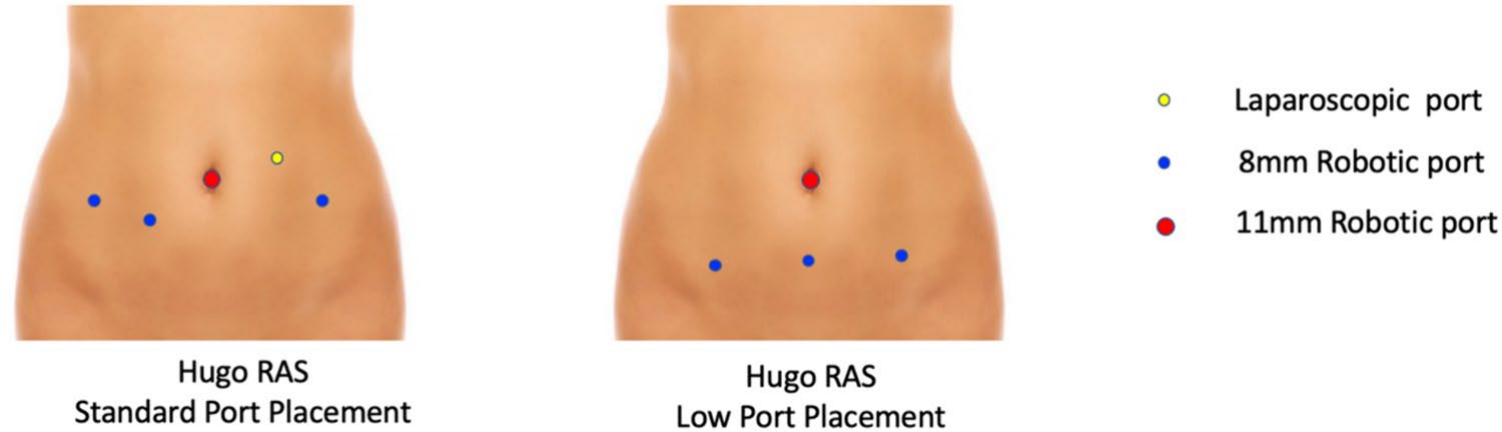
Port placement

The patient was placed in a supine low lithotomy position. Following general anesthesia, a Verres needle was inserted at the umbilicus to create pneumoperitoneum, followed by the introduction of an 11 mm robotic port through the umbilicus for the endoscope. A 3D 30-° scope was used. After achieving the Trendelenburg position, two to three additional 8 mm robotic ports were inserted, along with usually a 5 mm AirSeal port for an assistant. The choice between standard and low-port placement was determined based on the specific surgical procedure being performed (Fig. 1).

Arm positioning was performed by the surgeon and first assistant on each side of the patient. The arm that is furthest away is docked first. Tilt angles were preset on each of the robotic arms by the scrub nurse before being positioned next to the patient. The docking angle was set during the docking. The tilt and dock angles were determined based on arm placement.

**Fig. 2:**
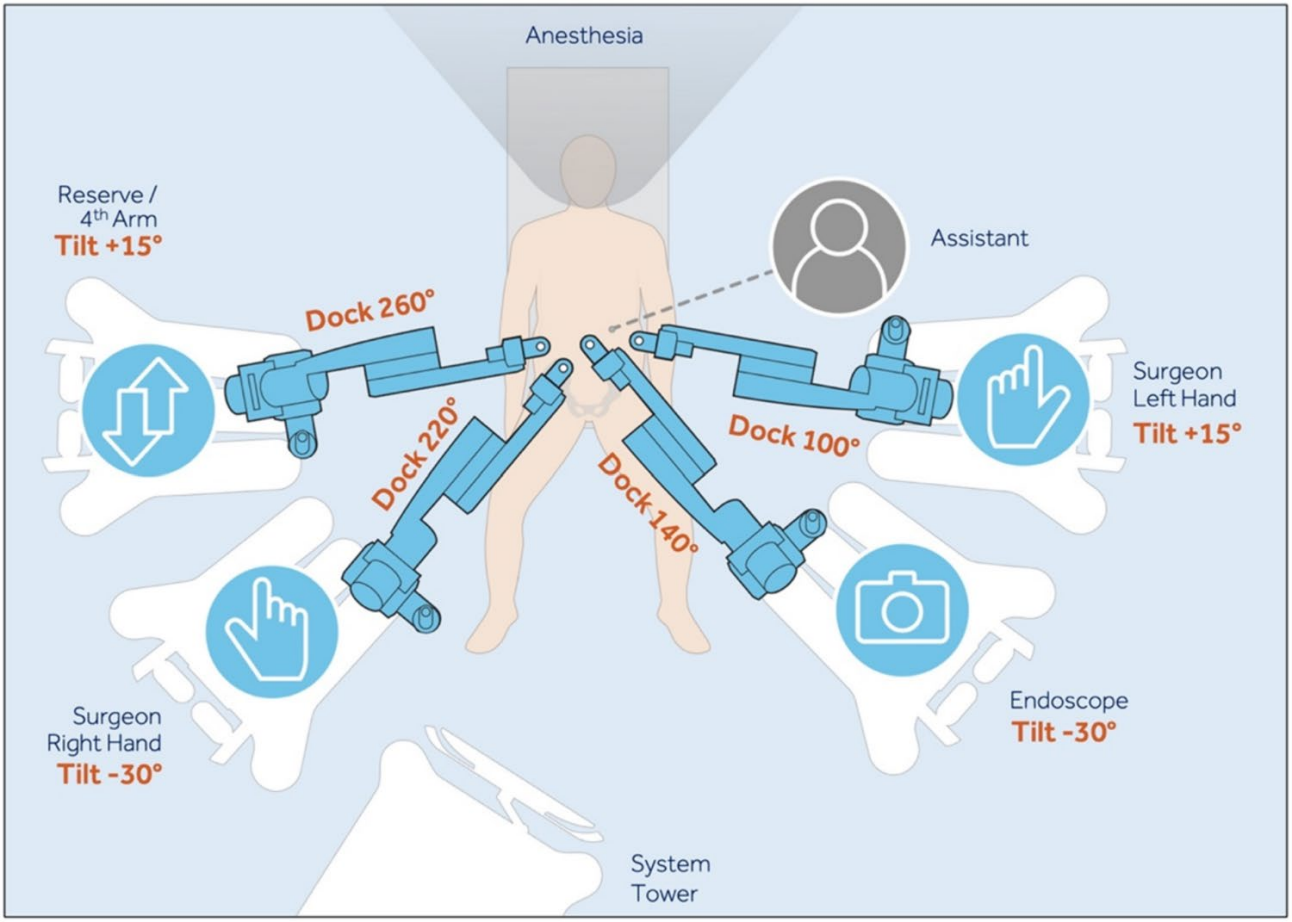
Symmetrical arm carts arrangement

“**Symmetric**” arm positioning included two arm positions on each side of the patient. The tilt of the top arms from both sides was configured to + 15°, and that of the lower arms on both sides was set to −30°. The clockwise angles from the left upper part to the right upper part were 100°, 140°, 220°, and 260°. The assistant port was inserted on the left side between Arms 1 and 2. (Fig. 2).

**Fig. 3:**
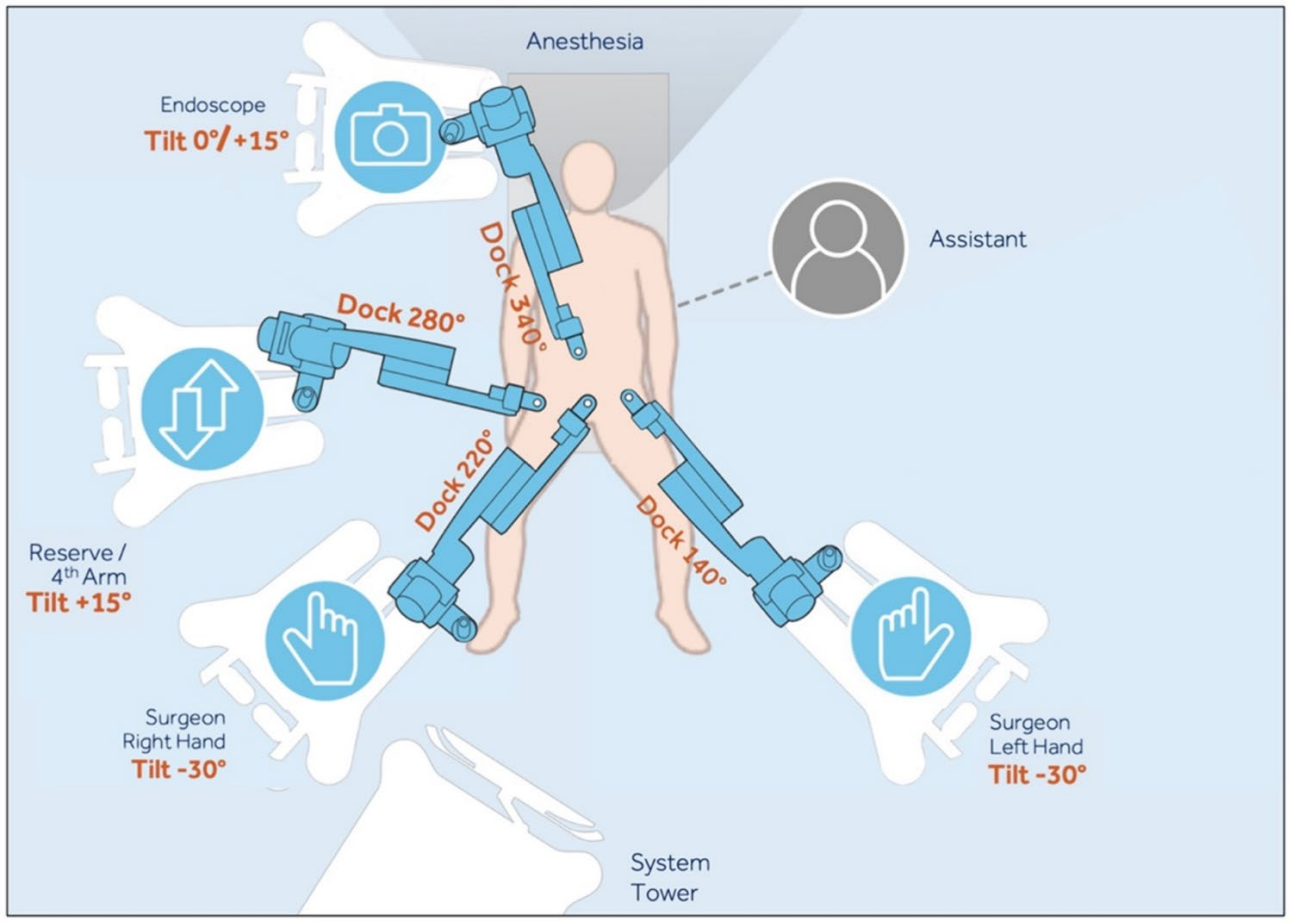
Asymmetrical arm carts arrangement

“**Asymmetric**” arm positioning included one arm positioned on the left side of the patient and three arms on the right side of the patient. Arm 1 had a tilt of −30° and a dock angle of 140°, arm 2 also had a −30° tilt and an angle of 220°, arm 3 had a tilt of + 15° with a dock angle of 280°, and the fourth arm was tilted to 0 / −15° with a dock angle of 340°. The assistant port was placed on the left side between arms 1 and 2 (Fig. 3).

Typically, our standard port configuration consists of four robotic arms with Symmetrical arm positioning, which we find works best for more complex cases. For more minor cases, particularly in young patients, we aimed for low-port placement with a reduced port configuration with only two robotic accessory ports and no assistant port. In such cases, we found that Asymmetrical arm positioning worked well, giving the assistant more room.

### Statistical analysis

Descriptive statistics were used to describe the parameters.

Categorical variables are summarized as numbers and percentages. For ease of reading, all continuous variables are reported as means and standard deviations, unless mentioned otherwise.

## Results

During the study period, 60 (17.2%) patients were operated by Hugo™ RAS system.

Table 1 provides an overview of the demographic data of the entire cohort. The surgeries performed using Hugo™ RAS included 32 (53%) endometriosis cases with stages ranging from 1 to 4, including two rectal shaving and one bowel resection, 16 (27%) hysterectomies, 6 (10%) adnexal surgeries, 3 (5%) myomectomy, one (2%) each of sacrocolpopexy, cervical cerclage and cesarean section scar defect repair.

**Table 1:** Demographic data of the Hugo™ cohort

Parameter		Population cohort *N* = 60
*Age (years)*		
Body mass index (BMI)		23.7 ± 5.8
Endometriosis total		32 (53%)
Stage I–II	24	
Stage III–IV	8	
	Segmental bowel resection (1), rectal Shaving (2)	
Adnexal surgery		6 (10%)
Cerclage		1 (2%)
CSSD repair		1 (2%)
Myomectomy		3 (5%)
Hysterectomy		16 (27%)
Hysterectomy + sacrocolpopexy		1 (2%)

Data are shown as number (%) or mean ± standard deviation, as appropriate

Table 2 presents the operative data, configuration data and satisfaction levels for the study cohort, including various surgical timelines. The mean port-placement time, defined from the first skin incision to the completion of port placement, was recorded at 13 min and 41 s (range 5–23). The mean docking time, measured from the end of port placement to the end of docking, averaged 5 min and 51 s (range 2–11). Mean console time, covering the entire operative period from the start of the main operation in the console to the end of surgery, was recorded as a mean of 1 h, 5 min, and 20 s (range 18–176).

**Table 2:** Operative data, configuration data and surgical team satisfaction for the study cohort

Parameter	Total time
Patient in the operation theater to first skin incision (min)	22:05 (15–44)
Port-placement time (min)	13:41(5–23)
Docking time (min)	5:51 (2–11)
Console time (hour)	1:05:20 (18–176)
Skin-closure time (min)	3:14 (2–7)
Symmetrical	22 (37%)
Asymmetrical	38 (63%)
Number of low ports cases	19 (31%)
Port changes due to troubleshoot per case	0.47 (± 0.6)
Number of arm repositioning due to clashing per case	0.75 (± 0.8)
Surgent satisfaction (1–10)*	8.4 (± 1.9)
Assistant satisfaction (1–10)*	6.9 (± 3.1)

port-placement time—time from first incision on the skin to the end of port placement

Docking time—time from end of port placement to the end of docking

Console time—operation time, from beginning of console operation to the end of the surgery

Data are shown as mean (range)

^*^On a scale of 1 to 10, where 1 was least satisfied and 10 completely satisfied

Data are shown as number (%), mean ± standard deviation or range, as appropriate

37% of cases used the “Symmetric” configuration, while 63% opted for the “Asymmetric” configuration. A total of ten cases required port changes due to troubleshooting, with an average of 0.47 changes per case (± 0.6). Arm repositioning occurred nineteen times, with an average of 0.75 adjustments (± 0.8). There were a total of 35 reported clashes, averaging 2.2 clashes per case (± 2.8). Surgeon satisfaction was rated at 8.4 (± 1.9) on a scale of 1 to 10, where 1 was least satisfied and 10 completely satisfied, while assistant satisfaction was reported at 6.9 (± 3.1), primarily due to space limitations and assistant arm collision.

**Fig. 4:**
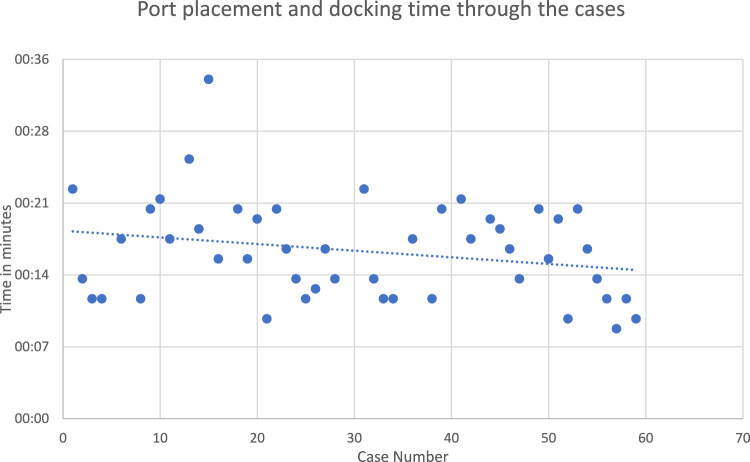
Time of port placement and docking time

Figure 4 illustrates time of port placement and docking time throughout the study period.

For patients in the Hugo™ RAS cohort, postoperative complications were monitored up until the of data collection. To date, no cases of intra-, peri- and postoperative complications at Clavien–Dindo Scale grade 3–5 were reported.

## Discussion

With experience in performing robotic surgery using both the da Vinci RAS and Hugo™ RAS systems, this study aims to share our early leaning in its application in advanced gynecological robotic surgery. In this article, we discuss the crucial aspects of port placement, arm configuration, docking time and essential insights for individuals new to the Hugo™ RAS system. We have detailed our experiences, learning curves, levels of satisfaction, and address specific challenges encountered. Despite the available data on the Hugo™ RAS, undisclosed aspects remain while the technology is still in the learning curve period.

Our primary outcome was to investigate the perioperative learning curves for port placement, arm positioning, docking and console time.

Pertaining to port placement, young gynecological patients and gynecologist alike, typically favor a low-port configuration for cosmesis, but it can also be relevant in some surgeries such as endometriosis wherein high port placement can be uncomfortable and is one of the inherent disadvantages of robotic surgery [17]. Studies comparing robotic-assisted surgery and laparoscopic surgery for endometriosis have primarily focused on factors such as surgery duration, postoperative outcomes, and precision [5, 18]. Here, we provide a different perspective on port placement. The da Vinci robotic system comprises four arms attached to a single boom, with one arm for the scope and three arms serving as accessory arms. In its default configuration, there are limits to how far these arms can be positioned from the scope and from each other [19]. In contrast, the Hugo™ RAS system’s modular arm design provides us with the flexibility to place the accessory ports in low port for needed cases such as endometriosis, without any constrain to the proximity to umbilical optical port [13].

Docking time has been an interesting topic of research for robotic surgery, with initial earlier publications reporting docking times ranging from 6 to 9 min [14, 15, 20]. As larger datasets were examined, they reinforced this trend [21]. In our study, the “Asymmetric” configuration was mainly utilized. We observed a docking time of 5 min and 51 s (with a standard deviation) range (2–11 min) for the Hugo™ RAS system, whereas for our team, the docking time with the da Vinci system typically was 2 min (range 1–8 min), this difference could be due to our learning curve with the docking of the Hugo™ RAS. Studies showed longer docking time with da Vinci then our report and almost similar to Hugo™ RAS system [22]. Our experience presented in this study indicates that a well-trained team with experience in robotic-assisted surgery can rapidly adapt to the docking process, as illustrated in Fig. 4 and supported in other manuscripts [23]. More insight regarding this issue, the modular nature of Hugo™ RAS arms, could give the impression that bringing in the four arms carts to the patient would prolong the docking process compared to the da Vinci system, where there is a single patient’s cart with four arms on it, but in practice that is not the case. With the da Vinci system, after placing the ports, surgeons need to wait as the da Vinci’s patient cart is driven over to the patient and have the laser guide aligned to the umbilical port. The endoscope arm is docked, and the endoscope is inserted and targeted before the remaining arms are docked. With the Hugo™ RAS there is no wait required as four individual carts are brought in while the ports are placed and immediately available for docking.

Our results shows that console time remained largely consistent between the two robotic systems. This observation further substantiates the notion of a rapid learning curve and highlights the absence of significant differences between the systems, particularly when utilized by well-trained surgeons.

The secondary outcomes of this study were focused on team satisfaction, system troubleshooting, arm repositioning, and complications at Clavien–Dindo Scale grade 3–4.

External clashes were encountered in earlier cases, which is one of the most common challenges in the system. Resolution of these events requires a clear understanding of how the arm cart operates. This situation can often be resolved by placing the arm carts apart, by changing the docking angle and / or tilt in one or both arms. Occasionally surgeons may be able to overcome an arm collision situation or limitation of the range of instrument reach without bedside assistance by modifying surgical techniques. This can be achieved by bringing mobile tissue more centrally, use of an alternate robotic arm or by pushing a tissue instead of pulling while still achieving the same desired tissue effect.

Despite the encouraging data, there are a few issues with the Hugo™ RAS system that deserve attention, as they can influence the satisfaction of surgeons. In cases such as endometriosis surgeries the use of bipolar and monopolar scissors, as well as forceps, mirrors the experience with the da Vinci system. However, in myomectomy procedures, the absence of a tenaculum, and the lack of a vessel sealer in cases of hysterectomy, may impact surgical satisfaction. While the needle holder remains the same, the absence of a suture cut feature is noticeable. Our understanding that these relatively minor issues will be improved upon in the near future.

Several other minor issues can contribute to discomfort during console time when using the Hugo™ RAS system. Notably, there is no indicator on tracking instruments that are out of sight. In addition, some inconveniences were noted, such as requiring a long press on the left side for switching between arms 3 and 4, and the double press needed to activate diathermy. Another issue we encountered was arm tremoroccurring when the robotic arm reaches its maximum range of movement, which can potentially impact surgical precision. In addition, we were unable to utilize our port-hopping technique due to the 10 mm scope, which cannot be easily transferred to other 8 mm ports. Consequently, when morcellation was required and access through one of the 8 mm ports became necessary, we had to employ a different scope for this purpose. These details highlight areas where refinement in the system can enhance the user experience.

In our comprehensive evaluation of our surgical team satisfaction while using Hugo™ RAS system, we considered all of the previously mentioned issues, including the occasional necessity for port changes due to troubleshooting and considered factors such as the low number of arm repositioning instances, as well as a relatively higher number of arm clashes. Despite these challenges, overall satisfaction of the lead surgeon remained high, with a rating of 8.4. This reflects a positive assessment of the system performance, even in the presence of certain operational issues.

Assistant satisfaction, while still favorable, was slightly lower. This can be attributed to the limited space available for the assistant in our arm configuration, which increases the risk of exposure to injuries resulting from arm movements [21]. In addition, there were instances where the assistant faced challenges when operating in the pelvic area, primarily due to arm occlusions and clashes. These factors, while not diminishing overall satisfaction, underscore areas for potential improvement in terms of safety and workspace optimization.

Another parameter for surgeon satisfaction is the ergonomic advantage that emerged through our experience with the Hugo™ RAS, which has been substantiated in other research papers, related to open console design [21]. Prior studies conducted with the da Vinci system highlighted certain shortcomings and potential eye discomfort [24], the open console design can lessen these issues, subsequently reducing eye fatigue and allowing for an upright sitting position, which, in turn, can alleviate muscle stiffness and discomfort and indeed allows the operator to sit or stand at the console as desired.

Finally, no complications at Clavien–Dindo Scale grade 3–4 were noted during the study period with all different surgical procedures, port placements and arm positioning.

This study has several strengths. First, it is one of the few investigations delving into the initial experiences with the Hugo™ RAS system, comprehensively addressing its advantages, limitations, and serving as a practical guide for newcomers to the field. Furthermore, the strength of the study lies in its singular centre-based approach, where all cases were managed consistently by a single surgeon, complemented by a proficient team of surgical assistants, nurses, and anesthetists, all of whom were well-versed in the realm of robotic surgery.

This study has some limitations. Most notably, the sample size was relatively small, potentially affecting the generalizability of the findings. Being drawn from a single institution, there may also be constraints on the diversity of surgeons that can learn from it.

## Conclusion

Our study offers insights into the realm of advanced gynecological robotic surgery, drawn from our diverse experience with both the da Vinci and Hugo™ RAS systems. We have documented our experiences, learning curves, levels of satisfaction, and addressed specific challenges faced during our journey highlighting key considerations that contribute to this learning process.

Our study contributes to the growing body of knowledge on advanced gynecological robotic surgery, emphasizing the need for ongoing exploration and refinement of the Hugo™ RAS system to maximize its potential and benefit surgeons and patients.

## Data Availability

The datasets generated and analyzed during the current study are available from the corresponding author upon request.
